# Digital Printing of Nanoenabled Enzymes on Textiles: An Integrated Antimicrobial and Antibiofilm Approach

**DOI:** 10.1021/acsomega.6c02477

**Published:** 2026-07-06

**Authors:** Guillem Ferreres, Garima Rathee, Tuser T. Biswas, Kristina Ivanova, Vincent A. Nierstrasz, Tzanko Tzanov

**Affiliations:** † Grup de Biotecnologia Molecular i Industrial, Departament d’Enginyeria Química, 16767 Universitat Politècnica de Catalunya (UPC-BarcelonaTech), Rambla de Sant Nebridi 22, Terrassa 08222 (Barcelona), Spain; ‡ Textile Materials Technology, Department of Textile Technology, Faculty of Textiles, Engineering and Business (including The Swedish School of Textiles), University of Borås, Borås SE-501 90, Sweden

## Abstract

Biofilm formation on textiles presents significant challenges in healthcare, industry, and daily use, contributing to microbial contamination, infections, and material degradation. Among biofilm-forming pathogens, *Pseudomonas aeruginosa* is particularly concerning due to its multidrug resistance and ability to evade conventional antibiotics, significantly increasing healthcare burden. To address this issue, we developed medical textiles digitally printed with multimodal silver–chitosan–acylase nanoparticles (AgCS@AC NPs). Fabrics endowed with antimicrobial and quorum-quenching properties inhibited bacterial growth and biofilm formation against *P. aeruginosa*. The enzyme acylase disrupted the quorum-sensing process of bacterial communication and prevented biofilm development, while the AgCS component of the NPs provided antimicrobial efficacy. Cytotoxicity assays confirmed that printed AgCS@AC NPs did not compromise human fibroblast or keratinocyte viability, ensuring biocompatibility. The eco-friendly, scalable, and versatile digital printing technology was innovatively validated for producing enzyme-enabled nanocomposite antimicrobial textiles for medical applications.

## Graphical abstract

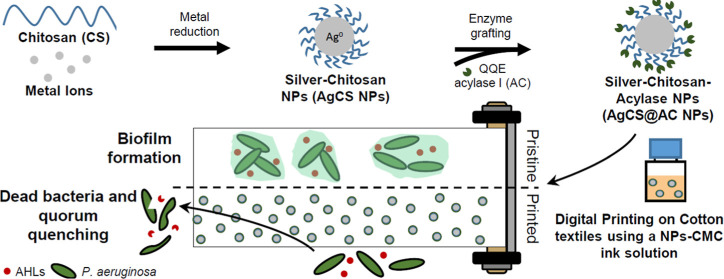

## 1 Introduction

Healthcare-associated infections (HAIs) remain a critical global health challenge, affecting approximately one in 31 hospitalized patients in the United States daily.^1^ The World Health Organization’s 2015 Global Action Plan on Antimicrobial Resistance (AMR) warned that AMR threatens “the very core of modern medicine”.^2^ In response, the 2024 UN General Assembly committed to reducing by 10% the estimated 4.95 million annual AMR-associated deaths by 2030.^3^ Among the most challenging pathogens, *Pseudomonas aeruginosa* epitomizes the multidrug resistance threat. Listed as a WHO priority pathogen for new antibiotic development,^3^ this opportunistic bacterium accounts for 7.5% of severe HAIs and over 25% of multidrug-resistant bacteraemia cases, with mortality rates exceeding 40% in critically ill patients and treatment costs averaging 40,000 US dollars per episode.^4,5^

The pathogenicity of *P. aeruginosa* is fundamentally driven by its sophisticated biofilm architecture, where bacterial communities encased in self-produced extracellular matrices confer transient protection against antibiotics, promoting resistance development.^6–8^ Biofilm tolerance to antibiotics is multifactorial, involving physical, physiological, and genetic determinants, with antibiotic resistance driven by mutations from repeated exposure to high levels of antibiotics.^7,9^ This biofilm development is governed through quorum-sensing (QS) systems mediated by *N*-acyl homoserine lactones (AHLs), enabling population-wide coordination of virulence factor expression and surface colonization.^10^ Recent advances in understanding QS mechanisms have revealed that disrupting these communication pathways, termed quorum quenching (QQ), offers a promising antivirulence strategy that circumvents traditional resistance selection pressure while targeting the root cause of biofilm-associated infections.^11–13^ QQ enzymes, particularly AHL acylases, have demonstrated the ability to chemically degrade signaling molecules, preventing signal reception and inhibiting biofilm formation across various bacterial species, including *P. aeruginosa*.^14^ Advanced nanoformulations incorporating acylase enzymes have shown remarkable synergistic effects, with nanohybridized gentamicin/acylase systems demonstrating 16-fold improved bactericidal activity and 97% attenuation of virulence factor production compared to conventional antibiotics.^15^

Nanoparticle (NP)-based antimicrobials have demonstrated significant promise in overcoming biofilm resistance, with studies reporting enhanced penetration capabilities and novel mechanisms of action.^16–19^ Layer-by-layer coating approaches have successfully integrated QQ enzymes with silver NPs, achieving synergistic bacterial eradication effects,^14^ while niosomes loaded with antibacterial agents have achieved effective targeting of intracellular pathogens.^20^ However, the integration of these advanced antimicrobial strategies with textile applications has been hindered by fundamental limitations in deposition technologies. Conventional methods, including dip-coating, spray-coating, and padding, suffer from critical deficiencies: heterogeneous distribution of antimicrobial agents resulting in variable antibacterial performance, substantial material waste, poor control over coating thickness and morphology, and, most critically, thermal and mechanical stresses that can denature sensitive bioactive components such as enzymes.^21,22^ These technical barriers have created a gap between laboratory demonstrations of antimicrobial efficacy and scalable manufacturing of clinically viable textile products, representing a significant bottleneck in addressing the HAI crisis.

Digital printing technology has revolutionized precision manufacturing across diverse sectors through its noncontact, digitally controlled deposition capabilities. In electronics, inkjet printing enables submicrometer placement of conductive inks for flexible circuits and organic photovoltaics with throughputs exceeding 1000 m^2^/h. In pharmaceutical manufacturing, digital printing facilitates personalized drug dosing and enables complex multidrug combinations in single dosage forms.^23^ Recent advances in textile biofunctionalization have demonstrated successful inkjet printing of enzymes like lysozyme and tyrosinase onto synthetic fabrics while preserving enzymatic activity through optimized ink formulations and printing parameters.^22,24^ Furthermore, digital inkjet printing has enabled uniform deposition of silver NP-based antimicrobials on cotton fabrics, establishing key protocols for NP textile functionalization.^25^ However, integrating QQ enzymes with antimicrobial NPs in digitally printed textiles remains an unexplored frontier with transformative potential against biofilm-associated infections.

Here, we report the first successful digital printing of QQ enzyme (QQE)-functionalized silver-chitosan NPs (AgCS@AC NPs) onto textiles, establishing a new paradigm for manufacturing active antimicrobial surfaces. Building upon established protocols for enzyme inkjet printing that preserve biological activity through controlled droplet formation and optimized ink rheology, this work advances the field by combining antimicrobial NPs with QQ activity.^22,24^ This approach integrates three synergistic mechanisms: silver NPs provide broad-spectrum antimicrobial activity through membrane disruption and reactive oxygen species generation, chitosan offers biocompatible film-forming properties coupled to inherent antimicrobial activity, and acylase enzyme disrupts bacterial communication by degrading AHL signaling molecules. The digital printing process reduces material waste significantly compared to conventional coating methods,^26^ preserves enzyme structural integrity through controlled droplet formation and gentle deposition,^27^ and enables precise spatial patterning with reproducible, scalable processing suitable for industrial manufacturing.^28^ Our multidisciplinary approach simultaneously tackles three critical healthcare challenges: reducing HAI incidence via enhanced antimicrobial efficacy, limiting AMR development through antivirulence targeting, and advancing sustainable manufacturing through waste reduction and process efficiency. We demonstrate that nanoenabled textiles fabricated using this method exhibit superior performance against both planktonic and biofilm-associated *P. aeruginosa* while maintaining biocompatibility with human fibroblast (BJ5tα) and keratinocyte (HaCat) cell lines. This work positions digital printing as a transformative platform for next-generation antimicrobial textiles, with immediate applications in surgical drapes, wound dressings, and personal protective equipment, thereby advancing the global efforts to combat AMR and improve patient outcomes.

## 2 Materials and Methods

### 2.1 Reagents

Chitosan (50 kDa, degree of deacetylation 86.7%) was obtained from Kitozyme (Belgium). Silver nitrate, acylase I from *Aspergillus mellus* (E.C. 3.5.1.14, 4.5% (w/w) protein content and 2.49 U/mg specific activity), acetic acid, Muller–Hinton broth (MHB), cetrimide agar, tryptic soy broth (TSB), *N*-hexanoyl-dl-homoserine lactone, and Dulbecco’s modified Eagle’s medium-high glucose (DMEM) were purchased from Sigma-Aldrich (Spain). 1-Ethyl-3-(3-(dimethylamino)­propyl)­carbodiimide (EDC), *N*-hydroxysulfosuccinimide (NHS), alamarBlue cell viability reagent, LIVE/DEAD viability/cytotoxicity kit for mammalian cells, and LIVE/DEAD BacLight viability kit for bacterial cells were obtained from Thermo Fischer Scientific (Spain). *P. aeruginosa* (ATCC 10145), human fibroblast (ATCC-CRL-4001, BJ-5ta), and keratinocyte (HaCaT) cells were purchased from the American Type Culture Collection (ATCC LGC Standards, Spain). *Chromobacterium* *violaceum* CECT 5999 (*C. violaceum* CV026), a mini-Tn5 mutant of the wild-type *C. violaceum*, was provided by the Spanish Type Culture Collection (CECT, Spain).

### 2.2 AgCS@AC NP Synthesis

AgCS@AC was produced following a protocol previously developed in our group.^29^ Briefly, chitosan (1% w/v) was dissolved in 300 mL of ultrapure water containing 1% acetic acid. After the complete dissolution of the polymer, 1 M NaOH was added to raise the pH of the solution to 5.5. Then, 200 mL of 2 mg/mL silver nitrate solution was added to the mixture and stirred at 500 rpm for 3 days at 80 °C to obtain AgCS NPs. The NPs were subsequently purified by washing with Milli-Q water, performing three successive cycles of centrifugation at 18,000 g for 40 min, followed by resuspension, to remove any unreacted silver ions and excess of the biopolymer. The obtained AgCS NPs were incubated under stirring at 500 rpm with 4 mg/mL of acylase at pH 4.8 for 30 min at room temperature. Then, 200 mM of EDC and 50 mM of NHS were added, and the reaction was conducted for 2 h. The AgCS@AC NPs were purified by centrifugation at 18,000 *g* for 30 min, washed twice with Milli-Q water, and resuspended in Milli-Q water.

### 2.3 AgCS@AC NP Characterization

The AgCS@AC NPs were analyzed spectrophotometrically in the 230–930 nm range using an Infinite M200 microplate reader (Tecan, Switzerland). Additionally, lyophilized chitosan, AgCS NPs, and AgCS@AC NPs were subjected to Fourier-transform infrared (FTIR) analysis, performing 64 scans over the 4000–650 cm^–1^ range, using a PerkinElmer Spectrum 100 spectrometer (USA). Baseline corrections and spectral normalization based on the absorbance maximum of intensity were carried out using the PerkinElmer Spectrum software. ζ-Potential of the NPs was measured using a Zetasizer Nano ZS (Malvern Instruments Inc., UK). The size and morphology of the NPs were determined by a transmission electron microscope (JEOL JEM-2100 LaB6, Japan) operating at 200 kV, following the application of 10 μL of the NPs onto SiO_2_ grids. Particle size distribution histograms were generated measuring the diameter of 200 particles for each sample using the software ImageJ 1.54g.

The silver content in AgCS and AgCS@AC NPs was quantified using inductively coupled plasma mass spectrometry (ICP–MS). Sample preparation involved mixing 50 μL of the NP suspensions with 500 μL of 20% nitric acid, followed by incubation at 100 °C for 60 min to ensure complete silver dissolution. The mixture was then diluted to a final volume of 5 mL with Milli-Q water to achieve a 2% nitric acid concentration. The samples were filtered to remove any remaining solids and analyzed using an Agilent Technologies 7800 ICP–MS system (Agilent Technologies, USA) calibrated with an internal standard of ^45^Rh and a standard curve of ^107^Ag.

### 2.4 Ink Preparation

NPs resuspended in Milli-Q water were converted into an ink solution by mixing with an appropriate viscosity modifier and surfactants to ensure continuous ink drop formation, ejection from the printhead, and spreading on textiles. Sodium carboxymethyl cellulose (CMC, *M*_w_ ∼90,000) viscosity modifiers were tested for compatibility with NPs. Triton-X 100 was used as a nonionic surfactant. Viscosity and surface tension were measured using a modular compact rheometer (Physica MCR, Anton Paar) and an optical tensiometer (Attension Theta, Biolin Scientific), respectively. Inks with a viscosity of 7–11 mPa·s at 20 °C, shear rate 10,000 s^–1^, and surface tension of 32–36 mN/m were obtained. The inks were stored at room temperature for a maximum of 5 days without changes in the colloidal stability.

### 2.5 Antimicrobial Characterization of the AgCS@AC NPs

The antibacterial activity of AgCS@AC NPs and the inks was assessed toward *P. aeruginosa*. Bacteria were grown overnight at 37 °C in MHB. Then, 50 μL of the NP solutions, in serial dilutions 1:2 using MHB medium, was mixed with 50 μL of the bacterial inoculum at an optical density at 600 nm (OD_600_) = 0.01 (∼10^5^–10^6^ colony forming units (CFUw)/mL) in 96-well polystyrene plates. In the case of the ink without NPs, the CMC solution was diluted using the same dilution factor as its NP-containing counterpart. The samples were incubated for 24 h at 37 °C with shaking at 230 rpm. The absorbance of the samples was measured at 600 nm before and after the incubation, and the bacterial growth for each was calculated as follows:

$$bacterial\;growth\left(\%\right)=100\cdot \frac{{Abs\;sample}_{t=24h}-{Abs\;sample}_{t=0h}}{{Absgrowth\;control}_{t=24h}-{Abs\;growth\;control}_{t=0h}}$$

The biofilm inhibition activity of the synthesized NPs and the inks was further assessed. Bacterial inoculum was prepared in TSB, diluted until an OD_600_ = 0.01, and incubated with the NPs or inks for 24 h at 37 °C in sterile Eppendorf tubes (250 μL of inoculum and 250 μL of ink). After 24 h, the biofilm was washed three times with sterile PBS to remove the loosely attached bacterial cells. Subsequently, 500 μL of sterile PBS was added to the Eppendorf tube. The tubes were vortexed for 3 min and sonicated in an ultrasonic bath for 15 min at 35% amplitude to break down the biofilm structure and release the embedded cells. The resulting suspension was plated on cetrimide agar and incubated at 37 °C overnight. CFUs were then counted to assess bacterial viability.

### 2.6 Printing of the Cotton Fabrics

A plain weave 100% cotton fabric (120 g/m^2^) was used for printing. To increase the surface adhesion of the printed NPs, the fabric was plasma-treated by using atmospheric pressure glow discharge equipment (PLATEX 600, Grinp, Italy). A combination of oxygen and nitrogen as feed gases (1 L/min of each) was used at 1.5 kW electrical power, 1 m/min fabric feed speed, and 1.5 mm interelectrode distance. Helium gas (1.5 L/min) was supplied along the plasma treatment to create an inert environment. A drop-on-demand piezoelectric inkjet printhead (Dimatix Sapphire QS-256/80, Fujifilm, USA) with 100 dots per inch resolution was used for printing. It was mounted on a custom-made printing platform manufactured by Xennia Technology (United Kingdom). Inks were printed on the fabric samples as a solid rectangle on an A4-size area containing about 10 mL of ink. The fabrics were then dried at room temperature for 1 h and were further characterized.

### 2.7 Characterization of the Printed Cotton Fabrics

The printed fabrics were analyzed by FTIR spectroscopy (PerkinElmer Spectrum 100 FTIR spectrometer, USA). Spectra were recorded over a spectral range of 4000–650 cm^–1^, with a resolution of 4 cm^–1^ and a total of 64 scans. The contact angle of the pristine and printed samples was measured by casting a 2 μL water droplet on their surface using a Drop Shape Analyzer (Krüss, Germany). The SEM images were taken using a field-emission scanning electron microscope (SEM) (Merlin Zeiss, Germany) operating at 3 kV and complimented with energy-dispersive (EDX) spectroscopy. A layer of gold was sputtered on the fabrics before the analysis. Ag 3d and N 1s, X-ray photoelectron spectroscopy (XPS) spectra of the fabrics were acquired at a pass energy of 25 eV under ultrahigh vacuum conditions (5.0 × 10^–9^ mbar), using a Phoibos 150 MCD-9 detector in combination with an XR50 magnesium anode source operating at 150 W (D8 Advance, SPECS Surface Nano Analysis GmbH, Germany). Finally, the release of silver from the material was assessed by incubating the printed textile in 1 mL of PBS at pH 7.4 in a gentle orbital shaking at 80 rpm. Samples were collected at 1, 3, 5, 12,24, 48, 16, 96, 144, and 168 h, and the extracted volume was replaced with fresh PBS. The silver content in the aliquots was determined using ICP as previously described.

### 2.8 Antimicrobial Activity of the Fabrics Printed with AgCS@AC NPs

The antibacterial activity of the printed fabrics was assessed by contact killing. *P. aeruginosa* was grown overnight at 37 °C in MHB. Then, a bacterial solution was prepared at OD_600_ = 0.00028 in PBS. Fabric samples (1 cm^2^) were submerged in 1 mL of PBS for 2 h, followed by plating the liquid on cetrimide agar, incubation at 37 °C overnight, and colony counting.

For the antibiofilm assay, the fabric samples were placed in 24-well plates with 1 mL of TSB containing *P. aeruginosa* at an OD_600_ value of 0.05 and incubated for 24 h under static conditions. After the incubation, the fabrics were washed three times using sterile PBS to remove the loosely attached cells. Each sample was transferred into a 15 mL sterile tube containing 2 mL of sterile PBS, vortexed for 3 min, and incubated for 20 min in a SONIC 6MX ultrasonic cleaner (James Products Ltd., UK) at 35% amplitude. The bacterial suspensions were serially diluted and plated on cetrimide agar to quantify the viable bacterial cells within the biofilm.

After the washing steps, printed and nonprinted fabric samples were incubated overnight in a PBS-buffered solution containing 2% (v/v) paraformaldehyde and 2.5% (v/v) glutaraldehyde. Then, the samples were dehydrated through a series of 10 min washings with increasing ethanol concentration: one washing with 50% (v/v) ethanol, three washings with 70% (v/v), followed by one washing of each fabric with 90% (v/v), 96% (v/v), and 100% (v/v) ethanol. Finally, the samples were gold-sputtered and examined using SEM (Merlin Zeiss) operating at 1 kV.

The QQ activity of the NPs was assessed using the violacein bioassay. A single *C. violaceum* CECT 5999 colony was incubated in Luria–Bertani (LB) medium at 27 °C and 180 rpm for 16 h. After the incubation, bacteria were mixed with LB soft agar to a final concentration of OD_600_ = 0.01. The medium was supplemented with kanamycin (25 μM), and 7 mL of this mixture was poured into previously prepared LB agar plates to grow a bacterial lawn. The fabric samples were placed on the bacterial lawn, and a single drop (7 μL) of 10 μM of *N*-acetyl homoserine lactone (AHL) was cast onto them. The plates were incubated at 27 °C for 16 h to allow the production of violacein. The intensity of the pigment was assessed using the software ImageJ.

### 2.9 Toxicity Assessment

The cytotoxicity of the fabrics was tested on human keratinocyte and fibroblast cell lines HaCaT and BJ5αt, respectively, using an indirect approach. Cells (1 × 10^6^ cells/well) were seeded into 12 mm Transwell plates with 0.4 μm pore polycarbonate membrane insert (Corning, USA), onto which the fabric samples (1 cm^2^) were placed. This setup prevented direct contact between the fabric and the cells, thereby minimizing mechanical disturbance that could lead to false-positive cytotoxicity results. After 72 h, the membrane inserts with the fabrics were removed, and the cell viability was measured using an alamarBlue assay kit. All results are reported as mean value ±standard deviation (*n* = 3). In parallel, 80 μL of PBS containing 0.1% (v/v) of calcein AM and 0.1% (v/v) of ethidium homodimer-1 were added to each well. The cells were incubated for 15 min in the dark and then observed using a fluorescence microscope (Nikon/Eclipse Ti–S, The Netherlands) to assess the live (stained in green) and dead (stained in red) cell populations.^30,31^

### 2.10 Statistical Analysis

All data are expressed as mean ±standard deviation (SD) (*n* = 3). Statistical analysis was conducted using one-way ANOVA, followed by Tukey’s post hoc test with GraphPad Prism Software version 5.04. Differences were considered statistically significant at *p* <0.05. Statistical significance is indicated as follows: * for *p* <0.05, ** for *p* <0.01, and *** for *p* <0.001. The statistical analysis for the tests with only two data sets was performed using a *t*-test for independent data.

## 3 Results and Discussion

### 3.1 Characterization of the AgCS@AC NPs

The formation of the AgCS@AC NPs and their stability within the printing ink was confirmed by UV–vis and FTIR spectroscopy. As expected, after the formation of the NPs, the FTIR spectrum of chitosan changed due to its oxidation upon reduction of Ag^+^ to metallic Ag^0^.^32–34^ The signal between 3600 and 3100 cm^–1^ decreased, followed by the appearance of bands at ∼2850 cm^–1^, ∼1550 cm^–1^, and ∼1380 cm^–1^; these changes may be associated with the interaction of the polymer with the metal and the oxidation of hydroxyl groups. The presence of acylase I, after inclusion of the enzyme into the hybrid NPs, was confirmed by the new peaks corresponding to amide I and II signals at ∼1650/∼1550 cm^–1^, respectively, indicating the presence of peptide bonds (Figure 1A).^35^ The formation of Ag NPs was confirmed by UV–vis spectroscopy, detecting the typical surface plasmon resonance (SPR) signal at 420 nm.^36^ This peak was observed after the NP functionalization with acylase I and in the AgCS@AC NPs–CMC ink (Figure 1B), suggesting that the NPs remain dispersed and colloidally stable in the printing ink. The final ζ-potential of the nanostructure was 28.6 ± 0.85 mV. Finally, TEM images of AgCS@AC NPs displayed nanostructures of approximately 35 nm in size (Figure 1C), with a layer of organic material corresponding to acylase I. After formulating the AgCS@AC NPs in CMC, the NPs presented no changes in size, morphology, or distribution (Figure 1D), indicating that the ink did not affect the stability of the nanostructures.

**Figure 1:**
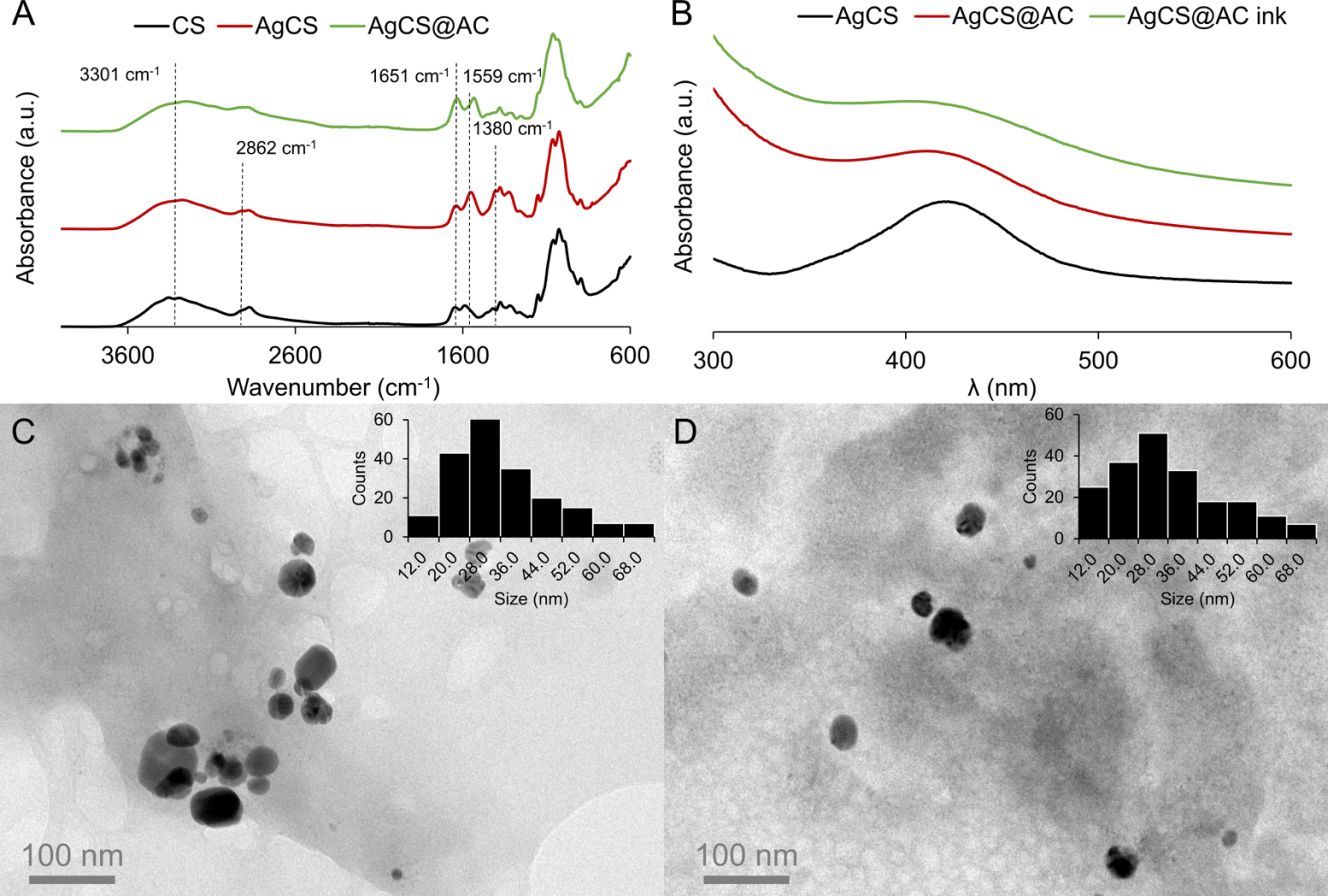
Characterization of the AgCS@AC NPs. (A) FTIR analysis of CS, AgCS, and AgCS@AC NPs. (B) UV–vis analysis of AgCS, AgCS@AC NPs, and AgCS@AC NPs in the CMC ink. TEM images of AgCS@AC NPs in water (C) and CMC ink (D). Insets: NP size histogram for AgCS@AC NPs in water dispersion (C) and the CMC ink (D).

### 3.2 Antimicrobial Activity of AgCS@AC NPs in the Printing Ink

The antimicrobial activity of the NPs dispersed in the ink solution was assessed by measuring the growth of *P. aeruginosa* after 24 h of incubation with the nanohybrids. The CMC ink without NPs displayed less than 20% bacterial growth inhibition, which is not considered an antimicrobial effect. On the other hand, AgCS@AC NPs in water and the CMC ink presented similar activity, completely inhibiting bacterial growth at a silver concentration of 7 ppm. Despite detecting low antimicrobial activity at 3.5 ppm of silver, the antibacterial performance of both formulations was similar and not affected by the CMC ink (Figure 2A). The antimicrobial activity of the AgCS core in these NPs has been widely studied, reporting the generation of ROS and the release of Ag^+^ that compromise important biological structures in bacteria, such as DNA and proteins.^37^ On the other hand, electrostatic interactions of chitosan with the bacterial membrane alter its permeability and potentially lead to its collapse.^38^

**Figure 2:**
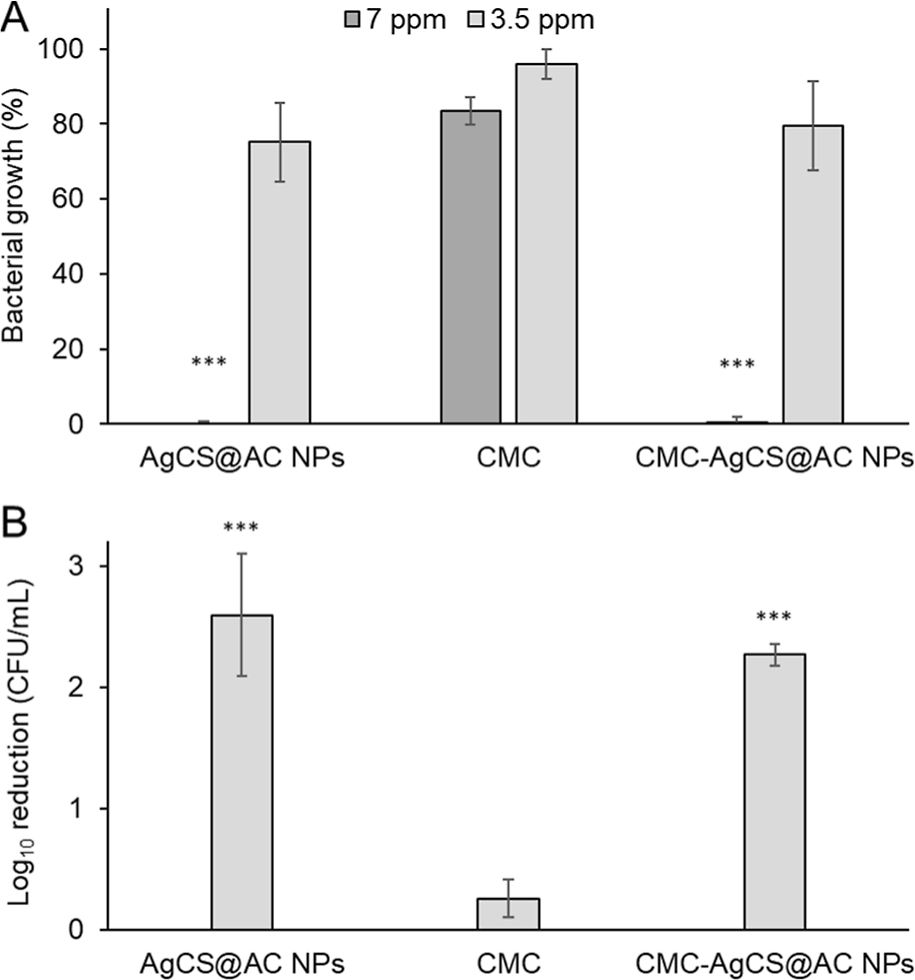
(A) Bacterial growth inhibition and (B) cell viability of *P. aeruginosa* in biofilms (expressed as log reduction values) evaluated in the presence of AgCS@AC NPs, CMC ink, and AgCS@AC NP-enabled ink. The log reduction values were calculated based on the initial bacterial concentration of 6 log_10_ CFU/mL. The CMC sample does not contain Ag and it has been diluted using the same dilution factor as its NP-containing counterpart. All data are expressed as mean ± SD (*n* = 3). *p* <0.05 was considered statistically significant, * for *p* <0.05, ** for *p* <0.01, and *** for *p* <0.001.

Similar findings were observed in the antibiofilm assays. Biofilm assessment is typically performed using the crystal violet assay, in which the crystal violet dye binds to the negatively charged components of the biofilm, including bacterial cells and extracellular polysaccharides, allowing for the quantification of the biofilm biomass.^39^ However, because of potential cross-reactions with the ink solution, colony counting of the viable biofilm cells was performed as an alternative. The performance of the NPs dispersed in the ink at a 3.5 ppm silver concentration was comparable to that of the water dispersion, reducing the amount of bacteria in the biofilm by approximately 2.2 logs. In contrast, the biofilm grown in the presence of *N*P-free ink contained the same number of bacteria as that in the control (Figure 2B). Biofilm formation is a QS-mediated process in *P. aeruginosa*. During this process, AHLs are released to activate the expression of the genes involved in biofilm synthesis. The acylase enzyme cleaves the amide bond between the acyl chain and the homoserine lactone ring in these signaling molecules, hindering the production of the biofilm extracellular matrix and reducing the attachment of bacteria on surfaces.^40^ Our previous work with these NPs demonstrated that acylase I is essential for AgCS NPs to inhibit *P. aeruginosa* biofilm formation.^29^ Therefore, the reduced bacterial presence in the biofilm observed in these results suggests that the enzyme is not significantly inhibited in the presence of the CMC ink.

### 3.3 Characterization of the AgCS@AC NPs Printed on Cotton Fabrics

The CMC ink was printed onto the cotton textiles without significant nozzle clogging or visible satellite drops, indicating good compatibility with the printhead used in the process. The ink uniformly covered the printed area immediately after deposition, confirming the effectiveness of both plasma treatment and the printing process. Subsequently, the printed textiles with the CMC ink and the fabrics were analyzed using FTIR spectroscopy to assess the presence of the NPs on the material. The sample with *N*P-containing ink exhibited new signals at 1650 and 1550 cm^–1^, corresponding to the stretching vibrations of C=O and amide –N–H groups, respectively, signals that were not observed in the pristine cotton. This confirms the presence of chitosan and acylase in the printed coatings. Additionally, the band around 2800 cm^–1^ could be assigned to the oxidized hydroxyl groups in chitosan formed during NP formation (Figure 3A).^29^

**Figure 3:**
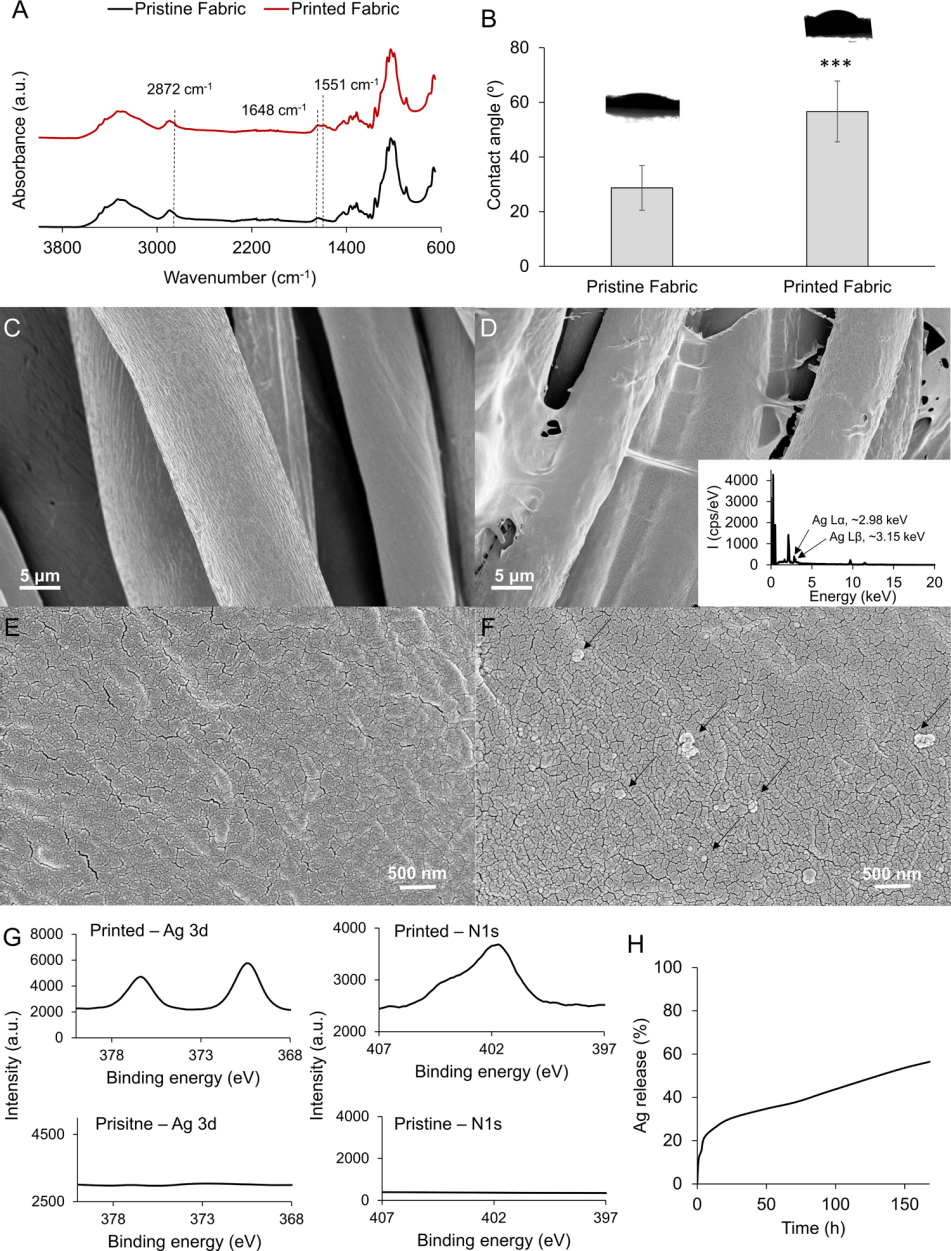
Characterization of the printed fabrics incorporating AgCS@AC NPs. (A) FTIR analysis of the pristine and printed samples. (B) Water contact angle of the pristine and printed samples All data are expressed as mean ± SD (*n* = 3). *p* <0.05 was considered statistically significant, * for *p* <0.05, ** for *p* <0.01, and *** for *p* <0.001. SEM images of the pristine (C,E) and printed (D,F) samples. Inset D: EDX spectrum of the printed textiles. (G) XPS spectra of Ag 3d and N 1s for the pristine and printed fabrics. (H) Silver release from the printed fabric incubated in PBS.

The successful deposition of AgCS@AC NPs on cotton fabrics was further confirmed by changes in surface wettability, assessed by water contact angle measurements. Pristine cotton showed values of 28.7°, consistent with its inherently high hydrophilicity. After printing with AgCS@AC NPs, the contact angle nearly doubled to 56.6°, indicating a shift toward more hydrophobic behavior. This increase could result in beneficial antibiofilm performance of the fabric, as a less hydrophilic surface may hinder initial microbial attachment, a key step in biofilm formation. Despite this shift, the fabric maintained an overall hydrophilic character, suggesting effective modification with AgCS@AC NPs (Figure 3B).

SEM analysis revealed notable differences between pristine and printed cotton fabrics. The untreated fabric exhibited a relatively smooth surface typical for the cotton fibers (Figure 3C and D). In contrast, printed fabric displayed different morphology, characterized by the presence of irregular nanostructures approximately 40–50 nm in size, distributed individually or forming small aggregates (Figure 3D and F). Additionally, the EDX spectrum showed characteristic peaks at ∼2.98 and 3.15 keV corresponding to the Ag Lα and Ag Lβ emission lines, respectively, confirming the presence of silver on the printed textile (Figure 3D inset). Notably, the cotton fibers in the printed fabrics appear partially embedded or “glued” together, unlike the separated fibers seen in the pristine sample (Figure 3C). This effect can be attributed to the CMC in the ink formulation, which acts as a viscous matrix that stabilizes the NPs during printing and improves their adhesion to the surface. Upon drying, the CMC forms a thin film or bridging layer between fibers, resulting in a fused or sticky network appearance. While this slightly alters the native fiber structure, it may enhance NP retention and improve the durability of the printed coating.

The successful deposition of the hybrid AgCS@AC NPs was also confirmed by XPS analysis. A comparison of spectra from pristine and printed fabrics revealed new signals in the printed sample at 370 and 376 eV corresponding to Ag 3d, along with a peak at 402 eV, attributed to the N 1s of chitosan and acylase (Figure 3G). Finally, the durability of the printed fabric was evaluated through the Ag release using ICP–MS. The initial amount of silver on the textile was ∼1.65 μg/cm^2^; following a 7 day incubation in PBS at pH 7.4, an initial burst release was observed with approximately 10% of the total silver load released within the first hour, probably corresponding to NPs weakly attached or adhered to the surface of the cotton fabric. By the end of the incubation period, approximately 45% of the silver remained on the fabric, demonstrating good retention and potential for sustained antimicrobial activity (Figure 3H). The observed stability is likely due to the combined effect of NP–fiber interactions and the digital printing process, which facilitates enhanced penetration and retention within the cotton matrix.^41^

### 3.4 Assessment of the Antimicrobial Properties of AgCS@AC NP-Printed Fabrics

To evaluate the antibacterial efficacy of the NP coating, the contact-killing and biofilm inhibition assays were performed comparing the pristine and *N*P-printed fabrics. In the contact killing assay, as expected, the pristine cotton fabric did not reduce the bacterial growth, while the NP-printed samples significantly reduced *P. aeruginosa* viability, achieving a ∼2.5 logs reduction after 3 h of contact with bacteria (Figure 4A).

**Figure 4:**
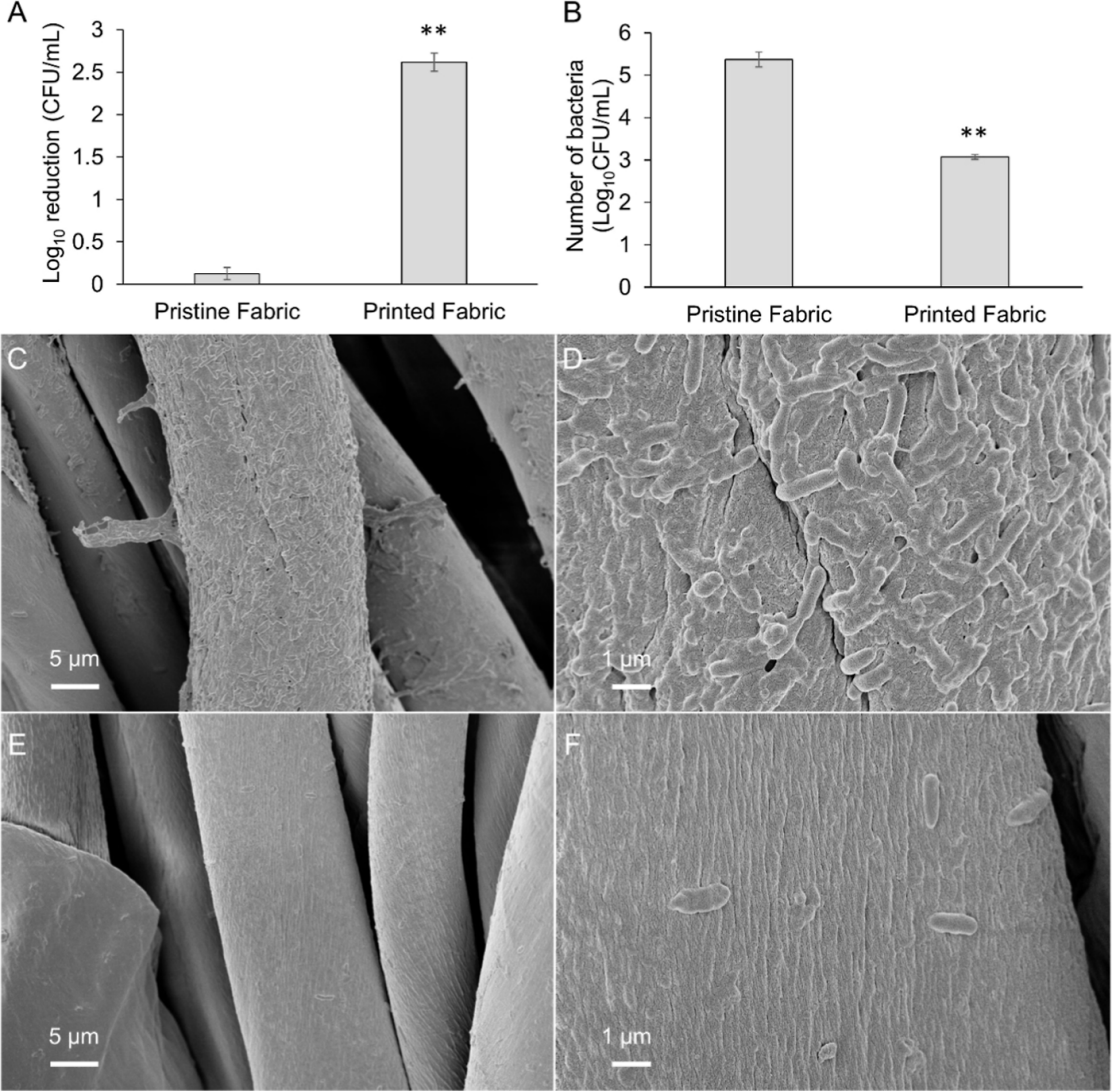
Antimicrobial activity of printed textiles with AgCS@AC NPs. (A) Evaluation of the antimicrobial activity of the pristine and printed fabrics. The log reduction values were calculated using an initial bacterial concentration of 3.63 log_10_CFU/mL. (B) Evaluation of the antibiofilm properties of the pristine and printed fabrics. All data are expressed as mean ± SD (*n* = 3). *p* <0.05 was considered statistically significant, * for *p* <0.05, ** for *p* <0.01, and *** for *p* <0.001. SEM images of *P. aeruginosa* on the pristine (C,D) and printed fabrics (E,F).

The biofilm inhibition capability of the AgCS@AC NP-coated fabric was also promising. Compared to the pristine cotton, the AgCS@AC NP-printed fabrics inhibited the *P. aeruginosa* biofilm formation, reducing the number of viable biofilm-forming bacteria on their surface by 2.3 logs (Figure 4B). These findings were further corroborated by SEM images (Figure 4D,F), which showed a dense population of intact, rod-shaped *P. aeruginosa* cells on the surface of pristine fibers, while the NP-enabled ones exhibited almost complete inhibition of biofilm formation. These antimicrobial and antibiofilm results illustrate the mechanism of action of the multimodal AgCS@AC NPs: (i) bacterial eradication by silver and chitosan and (ii) acylase quenching of the QS process, preventing biofilm formation.

Following the biofilm inhibition results, the textiles were subjected to a QQ assay to demonstrate that this bioactivity was due to the acylase degradation of the AHL signaling molecules. Fabric samples (1 × 1 cm^2^) were placed on LB agar plates inoculated with a *C. violaceum* CV026 strain, unable to produce AHLs by itself and requiring external supplementation of QS molecules to produce the violet pigment violacein.^42^ Then, AHL solution was drop-casted on the fabric. After incubation, none of the samples showed bacterial growth inhibition, indicating that the acylase in the AgCS@AC NPs was responsible for the QQ effect. As expected, the negative control of *C. violaceum* CV026, without AHLs, was not able to synthesize the violet pigment (Figure 5A). In contrast, bacteria with AHLs, as well as the pristine fabric, produced a clear violet halo (Figure 5B,C). The NP-printed fabrics, however, hindered the production of violacein (Figure 5D), being able to reduce the intensity of the pigment by 95% (Figure 5E). In a previous study, we demonstrated that AgCS NPs are not able to cleave AHLs, and only the functionalization with acylase I confers with QQ activity to the nanostructure.^29^ The results of that work indicate that acylase I is not deactivated within the NP system, and the present results further suggest that neither the ink nor the digital printing process significantly reduces the enzyme activity, allowing it to effectively catalyze the degradation of these QS molecules.

**Figure 5:**
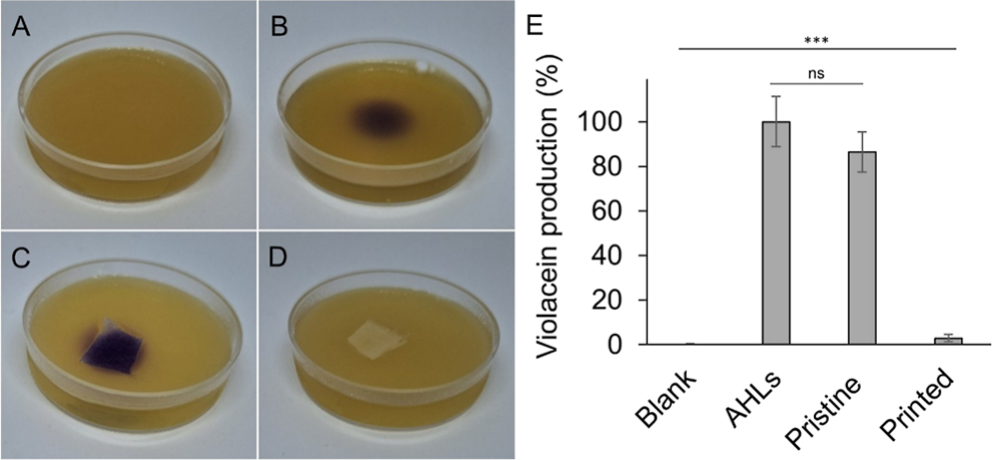
QQ assay. *C. violaceum* CV026 culturing in a plate without AHLs (A), with AHLs (B), and with AHLs in the presence of pristine (C) and printed (D) fabrics. (E) Assessment of the changes in the intensity of the color after the incubation with the fabrics. All data are expressed as mean ± SD (*n* = 3). *p* <0.05 was considered statistically significant, * for *p* <0.05, ** for *p* <0.01, and *** for *p* <0.001.

### 3.5 Biocompatibility Assessment

Potential toxicity of the NP-printed antimicrobial fabrics was tested on human skin fibroblast and keratinocyte cells. After 3 days of incubation, no difference in cell viability was observed between the samples incubated with pristine and printed textiles, as measured by the AlamarBlue assay,^43^ which evaluates cellular metabolism (Figure 6A). Furthermore, fluorescence microscopy revealed that skin cells exhibited comparable density, morphology, size, and viability when cultured on both pristine and printed fabrics (Figure 6B–E). The low toxicity of the AgCS@AC NP-printed fabrics is consistent with previous studies showing that incorporating biopolymers, such as chitosan, lignin, or hyaluronic acid, can mitigate the cytotoxic effects of silver NPs. In addition, these observations are consistent with the silver release data, which show that the cumulative release of AgCS@AC NPs after 5 days corresponds to approximately 0.67 ppm silver. This concentration is below levels reported to induce cytotoxicity in human skin cell lines and is consistent with our previous findings for these nanostructures.^29,44,45^

**Figure 6:**
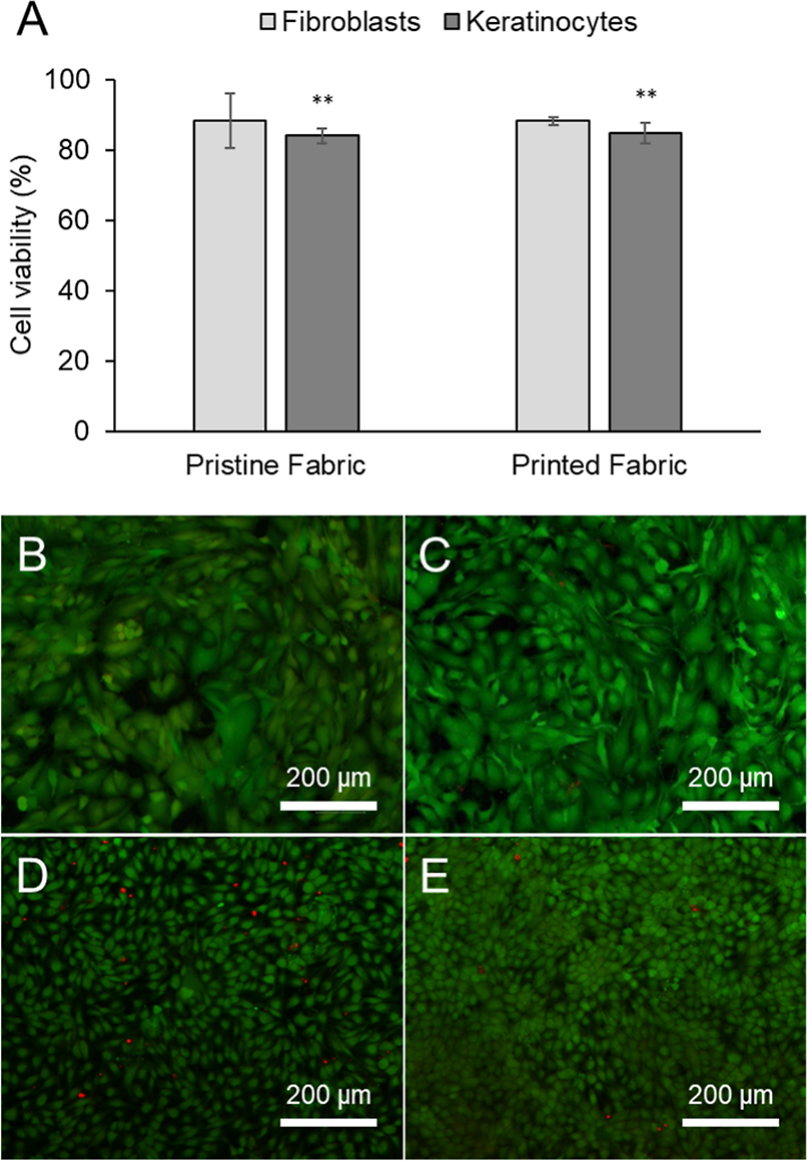
Cytotoxicity of the NP-printed fabric. (A) Assessment of the cellular metabolic activity of fibroblast (BJ5tα) and keratinocytes (HaCat) after 3 days of incubation with the pristine and printed textile using alamarBlue. All data are expressed as mean ± SD (*n* = 3). *p* <0.05 was considered statistically significant, * for *p* <0.05, ** for *p* <0.01, and *** for *p* <0.001. Fluorescence microscopy images of fibroblast (BJ5tα) and keratinocytes (HaCat) after 3 days of incubation with the pristine textile (B,D) and the printed textile (C,E).

## 4 Conclusion

The growing challenge of managing infections, driven by persistent biofilm formation and the spread of multidrug-resistant pathogens, underscores the urgent need for innovative antimicrobial strategies. Here, we report for the first time the successful digital printing of textiles with QQ enzyme-functionalized NPs that retain catalytic activity, conferring antibiofilm properties to the material. The digitally printed AgCS@AC NP-coated fabrics exhibited a dual-mode antimicrobial mechanism, achieving effective killing of planktonic *P. aeruginosa* while degrading bacterial QS molecules, suppressing virulence factors, inhibiting biofilm formation, and potentially reducing selective pressure. Furthermore, the integration of a biopolymer and enzyme into the silver-based nanomaterials produced a biocompatible hybrid system, showing no cytotoxic effects on human dermal fibroblasts or keratinocytes. Overall, these findings highlight the promise of enzyme-functionalized coatings applied on textiles via digital printing as a versatile platform for developing innovative, anti-infective materials.
